# Preferred physical characteristics of lidocaine thin film for women with vestibulodynia

**DOI:** 10.3389/fpain.2023.1217035

**Published:** 2023-09-18

**Authors:** R. Gina Silverstein, Martha Grace Cromeens, Caroline Rowland, Joseph A. Ogbansiegbe, Paul Mihas, S. Rahima Benhabbour, Erin T. Carey

**Affiliations:** ^1^ Department of Obstetrics and Gynecology, University of North Carolina, Chapel Hill, NC, United States; ^2^ School of Nursing, University of North Carolina, Chapel Hill, NC, United States; ^3^ Department of Health Policy and Management, Gillings School of Global Public Health, University of North Carolina, Chapel Hill, NC, United States; ^4^ Odum Institute for Research in Social Science, University of North Carolina, Chapel Hill, NC, United States; ^5^ Joint Department of Biomedical Engineering, University of North Carolina, Chapel Hill, NC, United States and North Carolina State University, Raleigh, NC, United States; ^6^ Division of Pharmacoengineering and Molecular Pharmaceutics, UNC Eshelman School of Pharmacy, University of North Carolina at Chapel Hill, Chapel Hill, NC, United States

**Keywords:** vulvodynia, vestibulodynia, localized provoked vulvodynia, dyspareunia, lidocaine

## Abstract

**Introduction:**

Vestibulodynia (VBD) is the most common cause of sexual pain in the United States, affecting up to 15% of reproductive-aged women during their lifetime with limited treatment options. The purpose of this study was to describe ideal physical characteristics of a vulvar film designed for insertional sexual pain in sexually active women with VBD.

**Methods:**

Twenty women were recruited to participant in one of six, semi-structured 60-minute focus group discussions regarding treatment options for VBD. Heterosexual women, aged 18–51 years old with a diagnosis of vulvodynia, vestibulodynia or insertional dyspareunia fit the inclusion criteria. Those who reported no episodes of vaginal intercourse in the prior 18 months were excluded. A new vulvar film technology loaded with 50 mg of 5% lidocaine was introduced to the group. Participants took part in focus groups on a rolling basis depending on availability. Focus group discussions were audio-recorded and transcribed verbatim. Two study investigators coded the transcripts using inductive coding and merged their respective projects to resolve disagreements. We analyzed data related to each code to develop code clusters and higher-level primary topics regarding device preferences. Data related to each of these primary topics was analyzed to assess the range of participant attitudes and preferences and to identify patterns within each primary topic.

**Results:**

One hundred and sixteen women were recruited, and twenty women were enrolled. The mean age for the participants was 33.3 years. Most women were educated with at least some college (93%), White (78.6%), married (75%), and had income greater than $100,000 (50%). Analysis of the focus group discussions identified five common topics addressed by participants: desired loaded medication, film size, film shape, film flexibility, and ease and accuracy of use. Concerns across topics included comfort, sexual spontaneity, and efficacy. Interest in loading the device with other acceptable medications or combination with lidocaine was independently noted in 2/6 (33%) of the focus groups.

**Discussion:**

Mucoadhesive vulvar thin films may be an acceptable drug delivery system for insertional sexual pain for women with VBD.

## 1. Introduction

Vulvodynia, defined as chronic vulvar pain for at least three months, is highly prevalent globally, with rates up to 15% in adult women (1, 2). Localized provoked vulvodynia, or vestibulodynia (VBD), is the most common presentation of this pain disorder, characterized by pain with palpation of the vulvar vestibule and/or attempted vaginal penetration. Vulvodynia has been shown to significantly impact patients' mental and physical health (3) and has large economic impacts, with estimates as high as $72 billion of direct and indirect costs annually in the United States (4).

The pathogenesis of VBD is complex, with microbial, immunological, hormonal, and genetic factors contributing to the heterogenous clinical presentation (5). On exam, inflammation and hyperesthesia is often noted within well-defined areas of the vestibule. Peripheral nerve sensitization, a pain mechanism described in VBD, is also considered a contributor to pain symptoms. At present, there is no consensus on treatment algorithms for vulvodynia, with limited strong, placebo controlled randomized clinical trials (2). Topical lidocaine is often used as first-line treatment due to low risk profile and accessibility. There is high level evidence that addressing pelvic floor dysfunction with pelvic floor physical therapy (PFPT) and psychosexual health are effective treatments. Topical hormonal medication and systemic medications (e.g., tricyclic antidepressants, calcium channel blockers) may have some efficacy (6–8) and surgical excision, while often considered the last resort, has shown significant symptom response in many patients (9). However, a recent meta-analysis of the available randomized control trials found no evidence of any treatment leading to improvement in dyspareunia, daily vestibular symptoms, or scores on a pain questionnaire (10). Inconsistent methods of pain assessment across studies and wide dosing ranges in prospective and randomized trials have contributed to the lack of strong evidence and standardized treatment algorithms (11).

While topical vulvar treatments are presently applied to the vulva in the form of gels, creams, and ointments, mucoadhesive thin films have been employed in vaginal drug delivery for years. On-demand contraceptives and spermicides are the most widely available vaginal thin films, preferred over semi-solid materials due to less leakage and mess, and better application and drug absorption (12–14). Regardless of indication, user acceptability of vaginal thin films is high and preferred over other topical drug delivery methods (15, 16). The vaginal thin film platform usability and acceptability offers a model for other genitourinary drug delivery systems. Our team has previously reported on the development of a mucoadhesive thin film as an alternate method to delivering lidocaine 5% to the vulva for localized, temporary pain relief for patients with VBD (17).

To explore perceptions and desires of treatments for VBD, a qualitative approach was used. Despite the widespread use of qualitative work in other chronic pain disorders, very few qualitative research has been performed in vulvodynia (18). The primary objective of this qualitative study was to describe the preferred physical characteristics of a mucoadhesive thin film drug delivery system for the local treatment of VBD symptoms in sexually active women. The perceptions of the film prototype size, shape, and current loaded drug were discussed with participants to understand acceptability and inform future design.

## 2. Materials and methods

Our study employed a blend of questionnaires and focus group discussion to understand the preferences for a vulvar film and the attitudes, beliefs, and motivations that influence these preferences (19). Focus group discussions with women with VBD were ideal in furthering understanding of an underrepresented topic: treatment preferences for VBD. The focus groups provided a less structured, participant-centered approach, activating “memory synergy” (20) which allowed participants to not only share their personal thoughts and preferences but to also recall experiences in response to shared–or differing–accounts from other participants (19, 21). The internal Institutional Review Board approved this study, number 21-0482. To understand the preferences of patients with vulvodynia, this study used focus groups to explore their thoughts on new treatments and to identify qualities they found important.

Participants were eligible for the study if they were heterosexual women, aged 18–51, with a history of provoked vulvodynia, vestibulodynia, or insertional dyspareunia. Women who reported no vaginal intercourse, or attempt at vaginal intercourse, within the last 18 months were excluded. Participants were recruited from a single, tertiary care pelvic pain center in the southeastern United States during presentation to a clinic visit or from self-response to a posted flyer. Respondents were screened by telephone to ensure they met study eligibility and recruited patients were scheduled for a group discussion on a rolling basis. Informed consent was obtained. They received $50 compensation for their time at study completion.

Focus group discussions were held from 11/2021 to 1/2022 over videoconference platform Zoom (Zoom Video Communications, San Jose, CA) in consideration of the COVID-19 pandemic and for scheduling ease. Due to the sensitive nature of the topic, groups were kept to 3–5 people, only first names were used, and participants could keep the video off if desired. A total of six, 60-minute semi-structed interviews were conducted by two research assistants who had been trained in facilitation of qualitative research interviews. An electronic consent and anonymous demographic and behavioral questionnaires were collected and managed using REDCap electronic data capture tools hosted at University of North Carolina (Research Electronic Data Capture, Nashville, TN) prior to the session.

**Figure 1:**
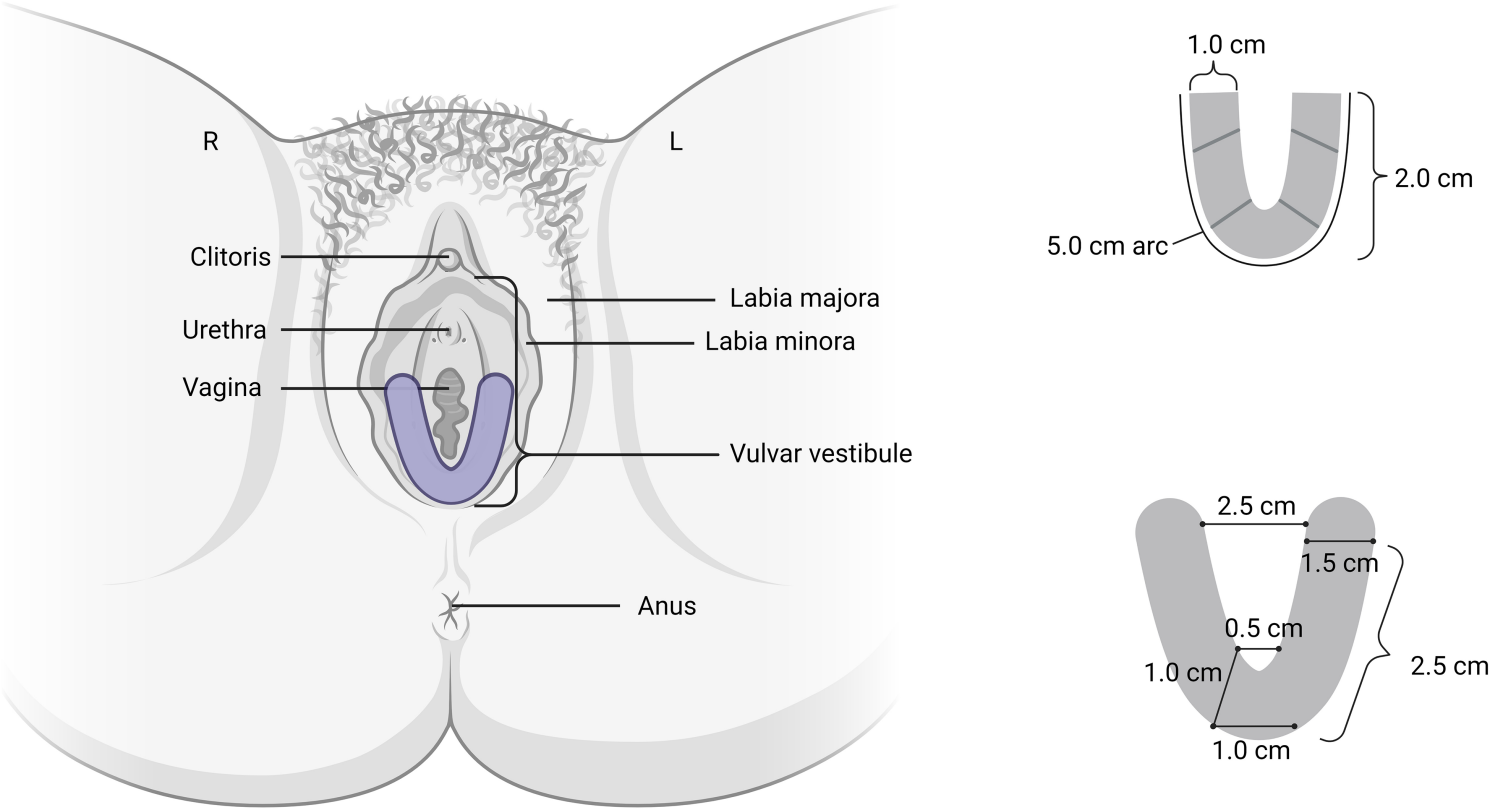
Prototype of vulvar film. Figure created with BioRender.com, accessed on 25 January 2023.

The moderator began with general open-ended questions about experiences with vulvodynia and treatment followed by a brief introduction of a new topical technology for the treatment of vulvodynia. Group discussion included general thoughts about the vulvar film, hypothetical vulvar film use, and desired characteristics of an ideal film. The drug delivery system was visually introduced to the participants. The film is a clear, flexible U-shaped mucoadhesive thin film designed for targeted drug dissolution into the vestibular tissue without residual liquid, cream, or gels (Figure 1). After drug dissolution, the film is then removed. Currently designed with 5% lidocaine, the local anesthetic effect is intended to last 60–90 min. All descriptions of vulvar film characteristic preferences were based on hypothetical rather than actual product use.

Audio from the focus groups was digitally recorded and transcribed verbatim. Two independent researchers reviewed each transcript and performed open and axial coding to assess codes and relationships among them (22, 23). The research team was trained by an experienced qualitative researcher (PM), who provided strategies for identifying, defining, and structuring codes using specialized qualitative software, MAXQDA (VERBI GmbH, Berlin, Germany). The training included strategies for resolving inter-coder disagreements, identifying meaningful code co-occurrences, and developing themes based on emerging patterns across focus groups. Reconciliation of discrepant codes were achieved through discussion between coders. The research team developed a posteriori or inductive codes driven by the focus group interviews (22). The inductive codes were based on identifying relevant preferences regarding the device, actions/experiences in relation to the device characteristics, and values regarding using a device, what mattered most to the participants, in their own words, and across groups. The coders identified primary topics which represented a condensed account of the participants' recurrent preferences. Primary topics were then individually examined to determine the breadth of responses and within-topic patterns. Any differences in interpretation were arbitrated by a third investigator.

## 3. Results

### 3.1. Study participants

Anonymous participant characteristics from self-administered online study questionnaires are described in Table 1. One hundred and sixteen women expressed interested in enrolling and screened by the research team. Of these, 57 did not respond to further communication, 15 were not eligible, 15 declined to participate, and 29 enrolled in the study and completed the self-administered online study questionnaires. Twenty of the 29 women continued to the focus group portion of the study. The mean age for the participants was 33.3 years and most women were educated with at least some college (93%), White (78.6%), married (75%), and had income greater than $100,000 (50%). A total of six focus groups were held with 3–5 participants each.

**Table 1:** Characteristics of the participants.

Variables	Results
Age, years—mean (SD)	33.3 (7.5)
Race—*n* (%)	
White	22 (78.6)
Black/African American	4 (14.3)
Other	2 (7.1)
Marital Status—*n* (%)	
Married/Living as Married	21 (75)
Never Married	7 (25)
Ethnicity—*n* (%)	
Not Hispanic/Latino	27 (96.4)
Hispanic/Latino	1 (3.6)
Education—*n* (%)	
High School	2 (7.1)
Some College	2 (7.1)
College	12 (42.9)
Post-Graduate	12 (42.9)
Income—*n* (%)	
Less than $20,000	1 (3.6)
Between $40,001 to $60,000	5 (17.9)
Between $60,001 to $80,000	4 (14.29)
Between $80,001 to $100,000	4 (14.9)
Greater than $100,001	14 (50)
History of lidocaine use for vulvar pain—*n* (%)	
Yes	18 (69.2)
No	8 (30.8)
Current use of lidocaine use for vulvar pain—*n* (%)	
Never	17 (65.4)
Sometimes	3 (11.5)
Occasionally	3 (11.5)
Most of the Time	1 (3.9)
All the Time	2 (7.7)
History of lidocaine use for pain with sex—*n* (%)	
Yes	13 (50)
No	13 (50)
Current use of lidocaine use for pain with sex—*n* (%)	
Never	18 (69.2)
Sometimes	2 (7.7)
Occasionally	1 (3.9)
Most of the Time	1 (3.9)
All the Time	4 (15.4)
When vulvar pain started—*n* (%)	
Less than 6 months	2 (7.7)
7 months—2 years	6 (23.1)
2–5 years	5 (19.2)
6–10 years	2 (7.7)
10 + years	11 (42.3)
Pain location—*n* (%)	
Clitoris	2 (6.9)
Urethral opening	5 (17.2)
Outer labia	5 (17.2)
Inner labia	11 (37.9)
Vaginal opening/vestibule	21 (72.4)
None of the above	1 (3.5)
When pain is experienced—*n* (%)	
Any time throughout the day	10 (34.5)
During non-sexual contact with the vulva	16 (55.2)
During sexual activity involving contact with the vulva	24 (82.8)
Other time	6 (20.7)

### 3.2. Primary topics

In discussing preferred physical characteristics of vulvar films, five major topics emerged: film medication, film size, film shape, film flexibility, and film application (Table 2).

**Table 2:** Illustrative quotes of participants in response to mucoadhesive vestibular drug-delivery thin film.

Primary topics	Illustrative quotes
Film medication Perceptions that lidocaine dose may affect treatment efficacy Film recognized as a possible delivery mechanism for other medications	I'm wondering …how much stronger the lidocaine would be vs. the creams that all of us—it sounds like all of us have put on, so if the lidocaine would be stronger. (FG1, P4) Definitely seems helpful for some people that really rely on lidocaine and benefit from it. (FG2, P1) Maybe like a topical neuroleptic…I mean, obviously, the lidocaine would help, but maybe gabapentin or Lyrica, something like that, that's more targeted. (FG4, P2) What I have to do now is I have to strategically put one leg over the bathtub and one leg over the other side of the tub, get a mirror, open everything up, put the lidocaine on a Q-tip and apply it specifically right at the opening…so that I don't get extra lidocaine on other stuff that I don't necessarily want numbed…if it gets into the wrong place or if it's not actually on where it needs to be, then it doesn't matter “cause it's numbed the wrong thing. (FG6, P2)
Film size Size elicits perceptions of product efficacy Desire for variety of sizes and widths	It would be nice to have different sizes…I would prefer if it were possible to order in, I don’t know, small, medium, like a different size. (FG4, P3) …a lot of my pain is at the entrance…if you can get this custom-made to your size. That would almost be the most ideal. (FG1, P1) It just seems like that is bigger than the area for me that I would want it to numb. (FG6, P2)
Film shape Shape impacts ease of application Shape impacts perceptions of efficacy Shape impacts perceptions of targeted drug delivery	Could it be used somewhere else? Specifically, if you have clitoral pain, or if you only have vulvodynia on one side… can it be used elsewhere? (FG4, P2) It's also just a U. I have some pain on the upper vestibule. I guess, potentially, I could use two, but I would be curious how that would go using on the upper. (FG2, P1)
Film flexibility Flexibility of the thin film related to perceptions of ease of application and comfort Edge sharpness and placement in skinfolds associated with discomfort	Everyone has very different size vaginas and vulvas, so there's not going to be a one size fits all, but if it's flexible and you can kind of put it where you need it to go…that's very helpful. (FG5, P3) If there's any way it's more flexible and you can bend it to the shape of your vestibule, it might be a little more helpful. (FG2, P2) I’d be worried about it being sharp on the edge. (FG5, P2)
Film application Minimal touching and long-lasting application preferred to minimize pain with application Easy applications ideal for a flexible lifestyle Thickness/thinness of film elicits perceptions of application ease Location application raises concerns about sanitation. Film delivery impacts ease of use and acceptability compared to current semisolid formulations (ointment, gel, creams)	…my biggest issue would be pain caused from the application itself…I anticipate it hurting. I know what I experience tryin’ to use tampons, tryin’ to put in my dilator, trying to do anything in that area. (FG3, P1) If you're out and about and you're gonna have a quickie or whatever you're doing, you could have these on the go, which that would be great. That would make people like us feel more, quote/unquote, “normal” if they were packaged to put in your purse and go wherever. I don't know. If it had less adhesive or whatever, that would be awesome. (FG1, P4) I think the thinner would be more malleable to be able to move where you would want it to go. Think that would be easier. (FG2, P3) I like that it's quick and then you can just remove it. There's not the residual cream hanging out there. (FG2, P1) The less messy sounds really, really good and appealing to me. (FG1, P1)

### 3.3. Film medication

Participants were introduced to the film with the planned delivery of lidocaine. Most participants (69%) had previously used lidocaine, and they shared their preferences and past experiences, prioritizing drug effectiveness, targeted application, and impact on sexual intercourse. Participants expressed interest in a medication that would alleviate pain with intercourse. As one participant stated, “I *would wanna be confident—or I’d want it to help*” (Focus Group (FG) 2, Participant (P) 1). Additionally, they discussed the need for focused application- desiring an anesthetic medication that would only be applied to painful areas without decreasing sensation in sensual/desired areas, a problem they avoided with previous experiences with lidocaine. Furthermore, they were concerned about lidocaine onset timing and how that impacts spontaneity, as well as their partners' experiences during sexual intercourse.

Across focus groups, participants shared positive and negative feelings regarding the use of lidocaine. Most patients voiced a willingness to try the film with lidocaine as the medication, but some questioned this option: “*Why are we just stuck on lidocaine?..To me, lidocaine is not the strongest thing out in the market*” (FG3, P3). They also were interested if other medications could be used in the same delivery device: “*Does it affect, if you numb things down there, does it affect arousal, orgasm? If you could use something like a gabapentin, Lyrica, something else that can be compounded would it be more beneficial for the experience,* vs. *just using something that*”*s going to numb the area?”* (FG4, P2)

### 3.4. Film size

Participants had a varied preference for film size. While some identified the proposed size as ideal, others would indicate the same size as too large or too small. They frequently voiced preference for multiple size options or customizable templates based on anatomy and pain characteristics.

### 3.5. Film shape

Similar to film size, the participants' perceptions of the film shape were influenced by their own anatomy and locations of pain. Varying shapes (e.g., V-, U-, or ovals with the ability to cut out a middle space) and widths were recommended to allow individualized coverage of the painful area. Regardless of individual preferences, the groups closely linked shape to ease of application, targeted drug delivery, and efficacy of the film.

### 3.6. Film flexibility

Flexibility, impacted by the film thickness and material, was a key concern among participants as related to optimal comfort, ease of movement, and better applications for different anatomy. “*This would be flexible? … if it's real flexible film, then that makes me less anxious to think about putting it on. I like the idea of it being on there to absorb in”* (Focus Group 1, P2) They worried about sharp edges pressing into sensitive areas. They wanted the film to be a thin, flexible material that could apply easily, form to their bodies, and have soft edges to prevent irritation or injury.

### 3.7. Film application

Participants had the most feedback around the process of film application. There were concerns around pain, allergies, lifestyle, quality of life, sex, and sanitation. One person said, “*Touching it hurts…but it still requires, I’m guessing, some level of firm pressure to apply. I know that would be painful. [Laughter] If the purpose is to avoid pain or to not have pain, then causing pain to not have pain seems counterproductive”* (FG3, P1). Other participants noted application benefits, including the device's portability and fast onset of action, facilitating lifestyle preferences and supportive of sexual spontaneity, a high priority across the groups.

The malleable properties were highlighted as pertinent to the ease of application and to allow for comfortable movement while in place. This was preferred over the messy and complicated applications of gels and creams. Recalling previous experiences, a participant said, “*my problem with lidocaine is that you put it on, and it's just haphazard that it numbs the wrong spots or it—honestly, it burns for me goin’ on, so it's not that great anyway. If you can apply it and it go into the right spots where it absorbs, that would be great”* (FG6, P3). They also liked that the application method would be less messy for their partners during sex and less likely to inadvertently numb their partner.

## 4. Discussion

User perceptions of targeted drug delivery systems for insertional sexual pain for patients with VBD are important in understanding the role of topical treatment and optimizing product design. In this qualitative study, a novel delivery system of local anesthetic was introduced to heterosexual, sexually active women with VBD. Most study participants reported an interest in trialing the vulvar thin film for temporary relief of VBD symptoms prior to penetrative vaginal intercourse. Some participants suggested a need for different shapes and sizes, dependent on the vestibule location where they experienced primary symptoms (e.g., upper vs. lower vestibule). There were also some concerns around the process of applying the film to an area that is painful to touch. Patients were interested in a new delivery method for lidocaine or other drugs that may improve some of the current application problems, such as imprecise dosing, messiness, and short duration of action. However, some were also apprehensive of trying a familiar medication despite a new platform, and expressed interest in the thin film drug delivery system loaded with other medications in addition to (or instead of) lidocaine.

Topical lidocaine is often a mainstay of treatment for VBD symptoms. While clinically not considered a long-term vulvodynia cure, local anesthetics are available and frequently used for short-term symptom management (24, 25). Topical application of lidocaine prior to a cotton swab test, also known as the “lidocaine test”, can eliminate mucosal allodynia from cotton swab rolling in patients with VBD (26). Repeated exposures to topical anesthetic treatment may desensitize the vestibule over time and provide long-term relief in some women (27). Recently, the combination of self-perineal massage plus topical lidocaine 2% improved perceptions of participants general health, vulvar pain, sexual function, and pain with vaginal penetration (28) The nightly application of lidocaine compared to weekly PFPT over 10 weeks each resulted in significant reduction in sexual pain in VBD, however the PFPT effect was greater than nightly lidocaine alone (29).

Some clinical limitations of topical lidocaine may be due to the delivery method—lidocaine cream, gel and ointments have a short drug retention time, are associated with messiness and leaking, and have imprecise dosing (16). As a result, desired therapeutic effects may not be achieved, resulting in poor patient adherence and dissatisfaction with use (14, 30, 31). This aligns with the patient reports in our study outlining mixed experiences with topical semisolid treatments, but an openness to trying a more targeted therapy with the potential for greater efficacy and less mess and leakage onto their partner.

We learned that participants were weighing multiple factors and considering several issues at the same time. The following quote demonstrates that participants are seeking a solution from several directions:

*I maybe find that the shape of it just seems off to me, based on what we talked about with the—being able to go internal. Then, yeah, I like that it's absorbing and no mess…as long as it's something that's really soft …I think it would be comfortable enough to put in.* (FG1, P3)

Prior and current painful experiences provided the foundation for participants' responses. Regardless, they all shared an optimism in finding a solution for their pain. A participant shared:

*When I heard about this trial, I was like, anything. I will try anything. I'm so sad to know that there are other people who are going through this, but it's so nice to hear you guys talking, to know that I'm not—sometimes, you start thinking, am I crazy?* (FG1, P4)

Participants also spoke extensively about the importance of intercourse and the perceptions and experiences of their partners. Future studies could explore the impact of different treatments on partners and try to gain their perspectives as well, as this was important to the participants with VBD. Many limitations of current topical medications for VBD can be addressed with a local, mucoadhesive drug delivery platform, however further research is needed to understand the priorities of patients with vulvodynia and acceptability of treatment options (16).

Our focus groups were performed via Zoom, preventing participants from holding, touching, and manipulating the prototype film, as only a limited quantity were created and could not be distributed to all participants. However, virtual focus groups are becoming more accepted as a valid method of qualitative research, with similar results to in-person focus groups (32). We also provided only one film shape; providing additional prototypes to allow comparisons may have elicited additional visual and tactile evaluations among potential users to better inform the product design. The demographic and sexual practice questionnaires obtained prior to the focus groups resulted in 29 women completing the on-line questionnaire, though only 20 women participating in the focus groups. To protect participants, the questionnaires were anonymous, however the study team was unable to identify participants who did not participate in the focus groups compared to those who completed the questionnaire alone. Finally, our focus group was limited to English-speaking participants from one geographic area, and the majority of participants were white and high socioeconomic status and may not be generalizable to the larger VBD population.

Despite qualitative methods considered necessary in describing social and relational domains of chronic pain, very few qualitative research has been performed in vulvodynia (17). Qualitative research can greatly contribute to guiding desired treatment for women with VBD. User perceptions and experiences of desired thin film properties demand further attention, especially as relevant to the development of novel platforms for VBD treatment. The current study provides insight into users' responses to key characteristics of loaded film medication, size, shape, flexibility, and application to guide future vulvar film design and other treatment options. This can lay the groundwork for much needed treatment development and future studies on evaluating clinical success of treatments.

In this study we aimed to explore user-relevant design parameters of prototype drug delivery systems. Perceptibility represents a critical set of factors driving preferred user characteristics. Women with VBD expressed an interest in a treatment platform with the potential for greater acceptability and efficacy compared to available topical vulvar regimens.

## Data Availability

The raw data supporting the conclusions of this article will be made available by the authors, without undue reservation.

## References

[1] Harlow BL Stewart EG. A population-based assessment of chronic unexplained vulvar pain: have we underestimated the prevalence of vulvodynia? J Am Med Womens Assoc. (1972) 58:82–8. 12744420

[2] Bergeron S Reed BD Wesselmann U Bohm-Starke N. Vulvodynia. Nat Rev Dis Primers. (2020) 6(1):36. 10.1038/s41572-020-0164-2 32355269

[3] Chalmers KJ Catley MJ Evans SF Moseley GL. Clinical assessment of the impact of pelvic pain on women. Pain. (2017) 158(3):498–504. 10.1097/j.pain.0000000000000789 28135211

[4] Xie Y Shi L Xiong X Wu E Veasley C Dade C. Economic burden and quality of life of vulvodynia in the United States. Curr Med Res Opin. (2012) 28(4):601–8. 10.1185/03007995.2012.666963 22356119

[5] Barnabei VM. Vulvodynia. Clin Obstet Gynecol. (2020) 63:752–69. 10.1097/GRF.0000000000000576 33074981

[6] van Beekhuizen HJ Oost J van der Meijden WI. Generalized unprovoked vulvodynia: a retrospective study on the efficacy of treatment with amitriptyline, gabapentin or pregabalin. Eur J Obstet Gynecol Reprod Biol. (2018) 220:118–21. 10.1016/j.ejogrb.2017.10.026 29202395

[7] Reed BD Caron AM Gorenflo DW Haefner HK. Treatment of vulvodynia with tricyclic antidepressants: efficacy and associated factors. J Low Genit Tract Dis. (2006) 10:245–51. 10.1097/01.lgt.0000225899.75207.0a 17012991

[8] Bachmann GA Brown CS Phillips NA Rawlinson LA Yu X Wood R Effect of gabapentin on sexual function in vulvodynia: a randomized, placebo-controlled trial. Am J Obstet Gynecol. (2019) 220:e1–89.e8. 10.1016/j.ajog.2018.10.021 PMC6310649 30365922

[9] Tommola P Unkila-Kallio L Paavonen J. Surgical treatment of vulvar vestibulitis: a review. Acta Obstet Gynecol Scand. (2010) 89:1385–95. 10.3109/00016349.2010.512071 20955094

[10] Pérez-López FR Bueno-Notivol J Hernandez AV Vieira-Baptista P Preti M Bornstein J. Systematic review and meta-analysis of the effects of treatment modalities for vestibulodynia in women. Eur J Contracept Reprod Health Care. (2019) 24(5):337–46. 10.1080/13625187.2019.1643835 31364893

[11] Bohm-Starke N Ramsay KW Lytsy P Nordgren B Sjöberg I Moberg K Treatment of provoked vulvodynia: a systematic review. J Sex Med. (2022) 19(5):789–808. 10.1016/j.jsxm.2022.02.008 35331660

[12] Green G Pool R Harrison S Hart GJ Wilkinson J Nyanzi S Female control of sexuality: illusion or reality? Use of vaginal products in south west Uganda. Soc Sci Med. (2001) 52(4):585–98. 10.1016/S0277-9536(00)00162-3 11206655

[13] Nel AM Mitchnick LB Risha P Muungo LT Norick PM. Acceptability of vaginal film, soft-gel capsule, and tablet as potential microbicide delivery methods among African women. J Womens Health (Larchmt). (2011) 20(8):1207–14. 10.1089/jwh.2010.2476 21774672

[14] Machado RM Palmeira-De-Oliveira A Martinez-De-Oliveira J Palmeira-De-Oliveira R. Vaginal films for drug delivery. J Pharm Sci. (2013) 102:2069–81. 10.1002/jps.23577 23649325

[15] Fan MD Kramzer LF Hillier SL Chang JC Meyn LA Rohan LC. Preferred physical characteristics of vaginal film microbicides for HIV prevention in Pittsburgh women. Arch Sex Behav. (2017) 46(4):1111–9. 10.1007/s10508-016-0816-1 27571742 PMC5332429

[16] Bunge KE Dezzutti CS Rohan LC Hendrix CW Marzinke MA Richardson-Harman N A phase 1 trial to assess the safety, acceptability, pharmacokinetics, and pharmacodynamics of a novel dapivirine vaginal film. J Acquir Immune Defic Syndr. (2016) 71(5):498–505. 10.1097/QAI.0000000000000897 26565716 PMC5040830

[17] Dahl DK Whitesell AN Sharma-Huynh P Maturavongsadit P Janusziewicz R Fox RJ A mucoadhesive biodissolvable thin film for localized and rapid delivery of lidocaine for the treatment of vestibulodynia. Int J Pharm. (2022) 612:121288. 10.1016/j.ijpharm.2021.121288 34800616 PMC8753993

[18] Mellado BH Pilger TL Poli-Neto OB Rosa e Silva JC Nogueira AA Candido dos Reis FJ. Current usage of qualitative research in female pelvic pain: a systematic review. Arch Gynecol Obstet. (2019) 300(3):495–501. 10.1007/s00404-019-05212-x 31201537

[19] Giacomini MK Cook DJ. Users’ guides to the medical literature: XXIII. Qualitative research in health care A. Are the results of the study valid? Evidence-based medicine working group. JAMA. (2000) 284:357–62. 10.1001/jama.284.3.357 10891968

[20] Kamberelis G Dimitriadis G. Focus groups: From structured interviews to collective conversations. (2013). 10.4324/9780203590447

[21] Patton MQ. Qualitative research & evaluation methods: Integrating theory and practice—michael quinn patton—google books. 4th ed. Thousand Oaks, CA: SAGE Publications, Inc (2015).

[22] Saldaña J. The coding manual for qualitative researchers. 4th ed. Thousand Oaks, CA: SAGE Publications, Inc (2021).

[23] Crabtree BF Miller WL. Doing qualitative research. 3rd ed. Thousand Oaks, CA: Sage Publications (2023).

[24] Schlaeger JM Glayzer JE Villegas-Downs M Li H Glayzer EJ He Y Evaluation and treatment of vulvodynia: state of the science. J Midwifery Womens Health. (2023) 68:9–34. 10.1111/jmwh.13456 36533637 PMC10107324

[25] Lamvu G Alappattu M Witzeman K Bishop M Robinson M Rapkin A. Patterns in vulvodynia treatments and 6-month outcomes for women enrolled in the national vulvodynia registry-an exploratory prospective study. J Sex Med. (2018) 15:705–15. 10.1016/j.jsxm.2018.03.003 29631955 PMC6613576

[26] Stenson AL Leclair CM Goetsch MF. Comparing vestibule examination techniques: light touch, serial forces, and the lidocaine test. J Low Genit Tract Dis. (2021) 25:236–42. 10.1097/LGT.0000000000000605 34016868

[27] Zolnoun DA Hartmann KE Steege JF. Overnight 5% lidocaine ointment for treatment of vulvar vestibulitis. Obstet Gynecol. (2003) 102:84–7. 10.1016/S0029-7844(03)00368-5 12850611

[28] Close A Culha MG Albert V Valancogne G. Exclusive manual perineal rehabilitation with lidocaine 2% gel in the treatment of provoked vestibulodynia: results from a single-arm interventional study. Int J Impot Res. (2023) 35(2):157–63. 10.1038/s41443-022-00537-9 35228685 PMC8884101

[29] Morin M Dumoulin C Bergeron SB Mayrand MH Khalifé S Waddell G 011 Efficacy of multimodal physiotherapy treatment compared to overnight topical lidocaine in women with provoked vestibulodynia: a bi-center randomized controlled trial. J Sex Med. (2016) 13:S243. 10.1016/j.jsxm.2016.04.011 26600287

[30] Johal HS Garg T Rath G Goyal AK. Advanced topical drug delivery system for the management of vaginal candidiasis. Drug Deliv. (2016) 23(2):550–63. 10.3109/10717544.2014.928760 24959937

[31] Caramella CM Rossi S Ferrari F Bonferoni MC Sandri G. Mucoadhesive and thermogelling systems for vaginal drug delivery. Adv Drug Deliv Rev. (2015) 92:39–52. 10.1016/j.addr.2015.02.001 25683694

[32] Keen S Lomeli-Rodriguez M Joffe H. From challenge to opportunity: virtual qualitative research during COVID-19 and beyond. Int J Qual Methods. (2022) 21:16094069221105075. 10.1177/16094069221105075 35692956 PMC9167989

